# Seroprevalence of Anti-*Echinococcus granulosus* Antibodies and Risk Factors for Infection in Blood Donors from Western Romania

**DOI:** 10.3390/life13040871

**Published:** 2023-03-24

**Authors:** Ana Alexandra Paduraru, Maria Alina Lupu, Rodica Lighezan, Radu Pavel, Octavian Marius Cretu, Tudor Rares Olariu

**Affiliations:** 1 Discipline of Parasitology, Department of Infectious Diseases, Victor Babes University of Medicine and Pharmacy, 300041 Timisoara, Romania; 2 Center for Diagnosis and Study of Parasitic Diseases, Department of Infectious Disease, Victor Babes University of Medicine and Pharmacy, 300041 Timisoara, Romania; 3 Patogen Preventia, 300124 Timisoara, Romania; 4 Clinical Laboratory, Institute of Cardiovascular Diseases, 300310 Timisoara, Romania; 5 Regional Blood Transfusion Center, 300737 Timisoara, Romania; 6 Discipline of Epidemiology, Department of Infectious Diseases, Victor Babes University of Medicine and Pharmacy, 300041 Timisoara, Romania; 7 Discipline of Surgical Semiology I and Thoracic Surgery, Department of Surgery I, Victor Babes University of Medicine and Pharmacy, 300041 Timisoara, Romania; 8 General Surgery Clinic, Municipal Clinical Emergency Teaching Hospital, 300254 Timisoara, Romania; 9 Clinical Laboratory, Municipal Clinical Emergency Teaching Hospital, 300254 Timisoara, Romania

**Keywords:** seroprevalence, human cystic echinococcosis, epidemiology, Romania, blood donors

## Abstract

Cystic echinococcosis is a worldwide-distributed zoonotic parasitic disease. This cross-sectional study aimed to assess the seroprevalence and risk factors potentially associated with *Echinococcus granulosus* in healthy blood donors from Timis County, an endemic region in Western Romania. Serum samples were collected from 1347 Romanian blood donors. Serologic tests to determine the presence of anti-*Echinococcus* antibodies were performed using an anti-*Echinococcus*-ELISA immunoassay. Anti-*Echinococcus* antibodies were detected in 38 blood donors, indicating an overall seroprevalence of 2.8%. The seropositivity rate was 3.7% in females and 3.1% in blood donors residing in urban areas. The highest seropositivity was found in the age group of 31–40 years (3.6%). There were no significant differences between *Echinococcus* seropositivity and gender, area of residence, age, contact with dogs, or raising sheep. This serologic survey evaluated for the first time the presence of *Echinococcus* antibodies in healthy blood donors from Western Romania and the potential risk factors associated with echinococcosis. Our results suggest that this zoonotic infection might evolve asymptomatically in apparently healthy individuals. Further studies should be conducted in the general population to estimate the true extent of human echinococcosis and its risk factors.

## 1. Introduction

Human cystic echinococcosis (CE) is a helminthic cosmopolitan zoonosis caused by the larval stage of *Echinococcus granulosus sensu lato* [1,2,3]. The World Health Organization (WHO) considers CE one of the most severe parasitic diseases in humans and ranks it second among the food-borne animal parasitoses. Moreover, CE has been listed among the 17 neglected tropical diseases [4] targeted for control or elimination by 2050 [5].

Human factors have allowed interaction between the sylvatic and domestic cycles of *E. granulosus,* which has led to the widespread perpetuation of this parasite in a variety of domestic and man-made life-cycle patterns. Therefore, cystic echinococcosis is the most important cestode zoonosis, with great public health and economic significance [6]. This parasitic disease is found mostly in agricultural and pastoral regions [1,3]. Increased rates of cystic echinococcosis were associated with poor hygiene and large-scale livestock rearing where dogs have access to offal from slaughtered animals [3,4,7]. The prevalence of *Echinococcus* infection increases with age, and women tend to be more frequently infected than men, perhaps due to domestic activities that bring them into closer contact with dogs [8]. High prevalences were reported in South America, Africa, China, the Middle East, the Mediterranean region, and eastern and southern Europe. In endemic regions, the annual incidence can reach up to 200/100,000 inhabitants [2,3,9]. Through comprehensive control programs, cystic echinococcosis has been eliminated in New Zealand, the Falkland Islands, Tasmania, Iceland, and Cyprus [8].

The life cycle of *E. granulosus* depends on predator–prey associations. Canids are definitive hosts (the adult cestode inhabits the small intestine), and livestock animals are intermediate hosts (harbor the larval stage of the parasite) [7,10]. Humans are considered accidental hosts and are, in most cases, not directly involved in the transmission of *E. granulosus* [10]. However, in unusual circumstances, humans can act as intermediate hosts: in Turkana (north-west Kenya, one of the highest endemic countries in the world), the cadavers of unburied nomadic people may be a source of infection for wild carnivores [11]. Humans become infected by ingestion of the parasite eggs shed in the feces of dogs (with contaminated food or water, or by direct contact with dogs) [12]. The eggs are infective upon release and can remain infective for up to a year depending on environmental conditions (can survive freezing but are sensitive to heat and desiccation) [8].

Cystic echinococcosis is characterized by the development of solitary or multiple cystic lesions [13]. Most patients (40% to 80%) develop a single cyst in a single organ. The liver is most frequently affected (70% of the cases), followed by the lungs (20%), and any other organ or structure (kidney, spleen, brain, pancreas, eye, testis, ovary, pleural, or abdominal cavities) [8,13,14]. Patients with CE can remain asymptomatic for 10 to 15 years, and this may explain why children represent only a small percentage of echinococcosis patients [8,10]. The incubation period of CE is highly variable. The growth rate of the hydatid cyst is still poorly understood and varies from 1 to 5 mm per year to 6 to 15mm per year. In 16% of cases, the cysts show no growth, or they collapse [8].

Clinical presentation in CE depends on several factors: the number and size of the cysts, the integrity of the cyst wall, the infected organ, the location of the cyst within the organ and its relation to surrounding structures, and the immune response of the infected individuals [10,12]. The role played by immune suppression in CE progression is suggested by the faster growth of hydatid cysts in patients with AIDS [10]. Moreover, the size of a hydatid cyst can be related to the genotype of *Echinococcus* [8]. In small or medium-sized cysts, symptoms will appear in the case of compression of vital structures. The rupture of a hydatid cyst or even minor fissures may cause severe complications (including life-threatening anaphylactic shock) due to antigen leakage [10,12].

Due to the absence of symptoms, the early stages of cystic echinococcosis are difficult to diagnose [1]. Imaging techniques (ultrasound, computed tomography, magnetic resonance imaging, and conventional chest radiography) are not only indispensable tools for diagnosing CE but also allow for establishing the localization and the specific stage [3]. The challenge for the imaging diagnosis is detecting cysts with a diameter of less than 2 cm. Ultrasound and X-ray are also used for population screening and follow-up. In endemic areas, mass population screening through ultrasound is considered the best method for the early diagnosis of CE [10].

Serologic tests aim to detect specific IgG anti-*E. granulosus* antibodies and have been regularly used as screening or confirmatory tests for diagnosis and follow-up, supporting the findings of imaging techniques [15]. The major antigenic source for immunodiagnosis is the hydatid fluid. In the case of early or inactive cysts, antigens are sequestered from the host’s immune system, and therefore negative serologic test results may be obtained. [8,10]. The polymerase chain reaction (PCR) technique is highly sensitive and reasonably specific due to its ability to identify the genus, species, and genotype *of Echinococcus* and distinguish them from other parasites [10]. Therefore, when possible, PCR is very helpful to confirm the diagnosis [3].

In patients with cystic echinococcosis, the prognosis is influenced by the pathogenicity of species/strains of *Echinococcus* [6]. The CE has a mortality rate of 2% to 4%, which may increase considerably in cases of inadequate care management [10]. The occurrence of post-surgery relapses of CE is estimated to be 6.5% [12].

Several seroepidemiological surveys assessed the seroprevalence of *Echinococcus* antibodies in the human population worldwide [16,17,18,19,20]. The seroprevalence of *Echinococcus* antibodies varies between countries, from 5.6% in Iran [16] to 6.5% in Sudan [17] and 6.9% in Turkey [18]. Cystic echinococcosis is considered an endemic parasitic disease in countries from eastern and southern Europe [21], including Romania, which is listed as a hyperendemic country by the World Health Organization (WHO). Only a few studies on the prevalence of *Echinococcus* have been recently conducted in Europe. Fotiou et al. reported a rate of 1.1% in the population from Central Greece [19], Żukiewicz-Sobczak et al. [20] reported a rate of 3.2% in forestry workers from eastern and southern Poland, and Lassen et al. [22] reported a rate of 3.3% in the Estonian general population.

To our knowledge, there are no scientific reports regarding the seroprevalence of anti-*Echinococcus* antibodies in the Romanian adult population. Therefore, in the present study, we aimed to determine for the first time the seroprevalence of anti-*E. granulosus* antibodies and risk factors for infection in healthy blood donors from Western Romania.

## 2. Materials and Methods

### 2.1. Study Area

**Figure 1:**
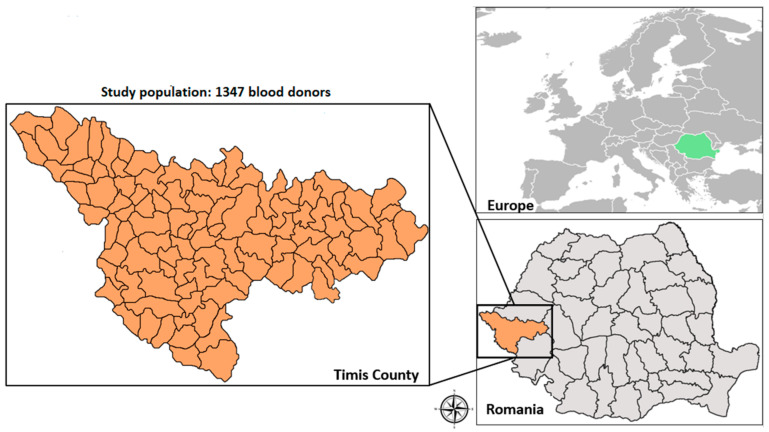
Study area map of Timis County.

The present study was conducted in Timis County, Western Romania (Figure 1). In 2018, the population was estimated at 750,512 inhabitants, with about 51.4% females and 59.3% residents of the urban area [23]. Timis County, the largest county in Romania with 8696.7 km^2^, is situated on the border with Hungary and Serbia and has three Romanian neighboring counties (Arad, Hunedoara, and Caras-Severin). The county territory includes all forms of relief, with the plain being more representative, covering the western and central parts of the county (6700 km^2^). The climate is temperate-continental, with Mediterranean and oceanic influences (warm summers and mild winters), making the cultivation of cereals and technical plants favorable [24,25]. According to the National Institute of Statistics, in the Agricultural Census performed in 2010, Timis County was the largest breeder of swine and sheep [25]. In 2018, 607,746 swine, 586,990 sheep, 41,184 cattle, and 18,037 goats were registered in Timis County [26].

### 2.2. Methods

Serum samples were collected from 1347 consecutive healthy blood donors who donated blood between 19 November and 21 December 2018 at the Regional Blood Transfusion Center in Timisoara. Every blood donor met the requirements for eligibility established by the Romanian Ministry of Health. Subjects with type I diabetes, schizophrenia, epilepsy, chronic hepatitis, liver cirrhosis, HIV, cancer, anemia, and asthma were excluded from the blood donation procedure [27]. During this study period, the subjects donated blood only once, and none of them had been diagnosed with cystic echinococcosis prior to the survey.

Venous blood samples (5 mL) were taken from all individuals who agreed to participate in the survey. Sera were stored at −20 °C until tested at the Center for Diagnosis and Study of Parasitic Diseases, Victor Babes University of Medicine and Pharmacy, Timisoara, Romania. Anti-*Echinococcus*-ELISA Ig-G kit (Euroimmun, Lübeck, Germany) designed for the EUROIMMUN Analyzer I-2P [28] was used for the detection of immunoglobulin G (IgG) antibodies to *Echinococcus*. The serologic test results were interpreted following the manufacturer’s recommendations: <0.8—negative; 0.8 to <1.1—borderline; and ≥1.1—positive [28]. For the purpose of this study, borderline serologic test results were considered negative.

All participants were asked to complete a questionnaire regarding the risk factors associated with *E. granulosus* infection. The information collected included demographic data (gender, area of residence, age, educational level, and occupational status) and personal behaviours potentially implied in acquiring the infection (smoking habits, household owning, dog ownership, and sheep raising). Study participants were grouped according to their age into 4 age groups: 18–30 years, 31–40 years, 41–50 years, and 51–63 years.

### 2.3. Statistical Analysis

Statistical analyses were performed using MedCalc for Windows, version 19.4 (MedCalc Software, Ostend, Belgium), and EPI Info v.7.2, CDC, Atlanta, GA, USA 2018. Data are presented as numbers (percentages), mean ± standard deviation (SD), and crude odds ratios (ORs) with 95% confidence intervals (95%CIs). Fisher’s exact test two-tailed or Chi-squared (χ^2^) test was used to assess differences between anti-*Echinococcus* positive and negative groups with respect to different criteria. A *p*-value < 0.05 was considered statistically significant.

This study was approved by the Ethics Committee of the Victor Babes University of Medicine and Pharmacy in Timisoara, and informed consent was signed by all participants. Each subject was informed regarding their serological test results.

## 3. Results

Of the 1347 healthy blood donors, 56.1% (755/1347) were females, and 72.7% (979/1347) were inhabitants of urban areas. The range of participants’ ages was between 18 and 63 years (mean age = 33.6 ± 10.9 years), with the age group 30–49 years being more representative (48.3%; 650/1347) (Table 1).

The overall seroprevalence of *E. granulosus* antibodies was 2.8% (38/1347; 95% CI: 2.06–3.85). The seroprevalence of *E. granulosus* antibodies in males and females was 3.7% (22/592) and 2.1% (16/755), respectively (Table 1). However, there were no associations between seropositivity and gender (*p* = 0.08; OR = 1.78; 95% CI: 0.92–3.42) (Table 2).

A higher seroprevalence was observed in people living in urban areas (3.1%; 30/979) compared to those from rural areas (2.2%; 8/368) (Table 1). However, it was not associated with *E. granulosus* seropositivity (*p* = 0.38; OR = 0.7; 95% CI: 0.31–1.55) (Table 2).

Seroprevalence tended to be higher in the age group 31–40 years (3.6%; 13/359) compared to the age group 18–30 years (2.3%; 14/607), age group 41–50 (2.9%; 8/272) and age group 50–63 years (2.8%; 3/109) (Table 1). Age was not associated with the presence of *E. granulosus* antibodies (χ^2^ = 1.44; *p* = 0.7) (Table 2). Educational level was not associated with the prevalence of *E. granulosus* of infection (Table 2). However, the seroprevalence tended to decrease with increasing level of education, from 6.7% in subjects who graduated primary school (2/30) to 2% in university graduates (13/638) (χ^2^ = 4.2, *p* = 0.28) (Table 1).

Occupational status, smoking habits, household owning, and dog ownership were not found to be associated with the prevalence of *E. granulosus* infection in blood donors (Table 2).

Regarding the potential animal-related risk factors, when analyzed together, animal raising was not associated with seropositivity (2.96%; 4/135; OR: 1.06; 95% Cl: 0.37–3.03; p=0.79). When the analysis was performed separately, anti-*Echinococcus* seroprevalence tended to be higher in individuals raising sheep (5.9%; 1/17; OR: 2.18; 95% Cl: 0.28–16.9) and cattle (3%; 1/33; OR: 1.08; 95% Cl: 0.14–8.11) compared to those raising swine, goats, or horses. Still, animal husbandry of any kind was not associated with seropositivity for anti-*Echinococcus* antibodies (Table 2).

## 4. Discussion

Cystic echinococcosis is a parasitic disease listed by the World Health Organization (WHO) as a Neglected Zoonotic Disease [4]. It is estimated that more than 1 million people worldwide are affected by this zoonosis. Even though any human can acquire the infection, the risk of infection is higher for animal handlers, dog owners, lab employees, and veterinarians [29]. High rates of infection are usually connected to sheep raising, pastoral areas, and farming [4].

Highly affected regions were reported in countries such as China, Peru, Argentina, Australia, Iran, Turkey, the Russian Federation, Italy, and Spain [30,31]. In Europe, the annual incidence of hospitalized individuals diagnosed with cystic echinococcosis varies from <1/100,000 inhabitants in continental France (0.42/100,000) [32] up to >8/100,000 in the Evora region [31] of Portugal (12.2/100,000) [33]. In Romania, high rates of infection were noted in both humans and animals [34,35]. The temperate climate, a high percentage of individuals working in the agricultural sector, and the existence of a large number of stray dogs ease the perpetuation of *Echinococcus* infection and may explain the data [36,37,38,39]. Cats seem to be resilient to harbor the adult worm of *E. granulosus*. However, they can act as intermediate hosts, and their coexistence with livestock animals might facilitate the spread of infection [40]. Moreover, there are documented situations in which certain fox species also contributed to the spread of the infection [6].

Serology is known to have lower sensitivity compared to imaging techniques. However, many serological surveys were performed worldwide to evaluate the prevalence and associated risk factors for *Echinococcus* infection [16,41,42]. Screening surveys based on serology are timesaving and permit a large serum sample analysis [18]. The seroprevalence in previously conducted surveys ranged from 0% in veterinarians from Poland [43] to 21.5% in the general population from Pakistan [1]

In the present study, the seroprevalence of specific anti-*Echinococcus* antibodies (2.8%) was lower than the seroprevalence found in healthy Iranian blood donors from Chaharmahal va Bakhtiari (3.3%) [44] and Fars provinces (5.6%) [16]. Although blood donors are not fully representative of the general population, testing for anti-*Echinococcus* antibodies in this population group allows for a large-scale assessment of echinococcosis prevalence and its risk factors. Higher seroprevalences of anti-*Echinococcus* antibodies were identified in studies conducted in the general population in Iran (3.96%) [45] and Turkey (6.9%) [18].

Our findings suggest a higher seroprevalence of anti-*Echinococcus* antibodies in females (3.7%) compared to males (2.1%), but the difference was not statistically significant. Our result is consistent with those reported by other authors [16,46]. Activities such as gardening, cleaning stables, or feeding and handling dogs increase the exposure of females to *Echinococcus* and may explain the difference [47].

It has been previously shown that cystic echinococcosis is mostly encountered in rural areas [7]. Interestingly, in our study group, anti-*Echinococcus* antibodies were more frequently detected in individuals from urban areas. No association between seropositivity to *Echinococcus* and area of residence was observed, similar to the results presented by Sarkari et al. [16] and Shafiei et al. [45]. The presence of stray dogs in the city suburbs may explain this result [24,48]. Moreover, it has been previously documented that stray dogs from urban areas represent a problem in Romania [38,39]. The high prevalence *of E. granulosus* infection in Romanian dogs [35,49] ensures the transmission of this zoonotic parasite to humans due to close contact [49] and contamination of soil and vegetables [45].

Cystic echinococcosis affects people of all ages [1], and its prevalence increases with age [7,50] due to increasing opportunities for older individuals to come in contact with eggs-contaminated matrices [30]. Whereas Sarkari et al. [16] found a higher seroprevalence in blood donors over 50 years, in our study the seroprevalence tended to be higher in the age group of 31–40 years. However, it is challenging to accurately assess the most affected age group due to the long prepatent period of the disease [36].

Educational level was not found to be significantly associated with anti-*Echinococcus* seropositivity in our survey. However, a low educational level was previously found to be associated with a higher risk of acquiring the infection [18,31]. Akalin et al. demonstrated that illiterate people and those with a low level of education had a higher rate of infection [18].

There was no association between occupational status and anti-*Echinococcus* seropositivity. However, the seroprevalence tended to be higher in retirees when compared to employee individuals or students, and this could be interpreted as increased exposure to the parasite related to aging. Similar findings were also reported by Ahmed et al. [17].

Similar to the results presented by Acosta-Jamett et al. [30] and Uchiumi et al. [7], smoking was not significantly related to infection in our study. However, the seroprevalence of anti-*Echinococcus* antibodies tended to be higher in blood donors with smoking habits compared to those who declared they do not smoke. According to Tamarozzi et al. [51], cystic echinococcosis is rather a “soil-transmitted” infection, acquired via a “hand-to-mouth” mechanism. One of the most significant modes of transmission for human cystic echinococcosis is the direct or indirect contamination of hands with parasitic eggs [52]. Smoking is a habit linked to hand-to-mouth transmission of *Echinococcus* (*s.l.*) and this may explain the difference [7].

Potential risk factors (household owning and dog ownership) investigated in our study were not significantly related to seropositivity for anti-*E. granulosus* antibodies. Previous studies reported similar findings [18,30,53]. However, Khabisi et al. [54] highlighted the significant role of dogs in the prevalence of infection. The proximity between households, combined with free-roaming dogs and inappropriate disposal of dog waste, seem to play a role in the transmission of the parasite [52].

In our survey, seropositivity tended to be higher in people involved in sheep and cattle husbandry, but without statistical significance. It has been previously documented that cattle and swine husbandry help maintain the life cycle of *Echinococcus* [55]. Goats, however, were reported to have a lower infection rate. An explanation could be the fact that they consume the upper portions of shrubs and plants, compared to cattle and sheep, who mostly eat ground grass that may be contaminated with infective eggs [56]. In horses, hydatid cysts are a rare finding, and usually small infertile caseous cysts are found incidentally [57,58].

This survey has several limitations. Blood donors are healthy adults from specific age groups (18–65 years) [59]. Individuals who tested positive for specific anti-*Echinococcus* antibodies had no signs or symptoms of cystic echinococcosis [16]. Detectable titers of anti-*Echinococcus* antibodies depend on multiple factors including localization, size, and the number of hydatid cysts. Usually, no antibodies are present in intact, small, or calcified cysts [19]. The transfusion center where the donors were enrolled is located in an urban area, which made participation more accessible to the urban population. This is also shown in the distribution of this study group’s members based on their place of residence. Hence, there could be a bias related to the higher seroprevalence in participants from the urban area compared to the rural one. Regarding age, our sampling included less represented ages of above 49 years, which could have induced a lower seroprevalence of anti-*Echinococcus* antibodies in this age group. Though our sample size was large, the number of individuals who tested positive for anti-*Echinococcus* antibodies was low, and this may also represent a limitation of this study in evaluating the risk factors associated with this infection. In addition, the incubation period of CE is long (lifelong in most cases) and poorly understood. Therefore, evaluating the risk factors may be challenging [47].

## 5. Conclusions

This survey provides new and important seroepidemiological data, evaluating the seroprevalence and potential risk factors associated with the presence of *Echinococcus* antibodies in blood donors. Results of the present study suggest that this zoonotic infection may be detected in healthy, asymptomatic individuals residing in an endemic region. Further studies should be performed on the general population to estimate the true extent of human echinococcosis in Romania.

## Figures and Tables

**Table 1:** General characteristics and descriptive statistics of blood donors from Western Romania ascertained by questionnaire.

Variables	Number of Tested Individuals (n = 1347)	%	Number of Individuals with Detectable Anti-*Echinococcus* IgG Antibodies (n = 38)	%
**Gender**				
Female	592	43.9	22	3.7
Male	755	56.1	16	2.1
**Area of residence**				
Rural	368	27.3	8	2.2
Urban	979	72.7	30	3.1
**Age group**				
18–30	607	45.1	14	2.3
31–40	359	26.7	13	3.6
41–50	272	20.2	8	2.9
51–63	109	8.1	3	2.8
**Educational level**				
Primary school	30	2.2	2	6.7
Gymnasium	174	12.9	7	4.0
Highschool	505	37.5	16	3.2
University	638	47.4	13	2.0
**Occupational status**				
Non-employed	167	12.4	6	3.6
Employed	869	64.5	27	3.1
Student	291	21.6	4	1.4
Retiree	20	1.5	1	5
**Smoking**				
Yes	429	31.8	14	3.3
No	918	68.2	24	2.6
**Household owning**				
Yes	395	29.3	12	3.0
No	952	70.7	26	2.7
**Owning dogs**				
Yes	327	24.3	9	2.8
No	1020	75.7	29	2.8
**Raising sheep**				
Yes	17	1.3	1	5.9
No	1330	98.7	37	2.8
**Raising swine**				
Yes	121	9.9	3	2.5
No	1226	90.1	35	2.9
**Raising goats**				
Yes	20	1.5	0	0
No	1327	98.5	38	2.8
**Raising cattle**				
Yes	33	2.4	1	3
No	1314	97.6	37	2.8
**Raising horses**				
Yes	15	1.1	0	0
No	1332	98.9	38	2.9

**Table 2:** Risk factors for infection in blood donors tested for the presence of anti-*Echinococcus* antibodies.

Potential Exposure to Risk Factor	Individuals with Detectable Anti-*Echinococcus* Antibodies		Individuals without Anti-*Echinococcus* Antibodies		OR (95% Cl)	*p*-Value
	Exposed	Non-Exposed	Exposed	Non-Exposed		
**Gender**						
Female	22	-	570	-	1.78 (0.92–3.42)	0.08
Male	16	-	739	-		
**Area of residence**						
Rural	8	-	360	-	0.7 (0.31–1.55)	0.38
Urban	30	-	949	-		
**Age group**						
18–30	14	-	593	-	Ref.	
31–40	13	-	346	-	0.63 (0.29–1.35)	0.23
41–50	8	-	264	-	0.78 (0.32–1.88)	0.58
51–63	3	-	106	-	0.83 (0.24–2.96)	0.73
**Educational level**						
Primary school	2	-	28	-	Ref.	
Gymnasium	7	-	167	-	1.7 (0.34–8.63)	0.62
Highschool	16	-	489	-	2.18 (0.48–9.97)	0.26
University	13	-	625	-	3.43 (0.74–15.96)	0.14
**Occupational status**						
Non-employed	6	-	161	-	Ref.	
Employed	27	-	842	-	1.16 (0.47–2.86)	0.81
Student	4	-	287	-	2.67 (0.75–9.62)	0.18
Retiree	1	-	19	-	0.7 (0.08–6.2)	0.55
**Smoking**	14	24	415	894	1.25 (0.64–2.45)	0.50
**Household owning**	12	26	383	926	1.11 (0.55–2.23)	0.76
**Owning dogs**	9	29	318	991	0.96 (0.45–2.06)	0.93
**Raising sheep**	1	37	16	1293	2.18 (0.28–16.9)	0.39
**Raising swine**	3	35	118	1191	0.87 (0.26–2.86)	1
**Raising goats**	0	38	20	1289	0 (NA)	1
**Raising cattle**	1	37	32	1277	1.08 (0.14–8.11)	0.61
**Raising horses**	0	38	15	1294	0 (NA)	1

NA: not applicable; OR: odds ratio; 95% Cl: 95% confidence interval, Ref.: Reference.

## Data Availability

Data are available upon request.
